# The Prevalence of Poor Behavioral Health Among University Students by Gender, Sexual Orientation, and Racial Identity: The Role of Discrimination and Microaggressions

**DOI:** 10.3390/ijerph23060776

**Published:** 2026-06-09

**Authors:** Tolulope M. Okuneye, Andrew J. Godley, Elaine C. Russell, Lisa L. Lindley, Kenneth W. Griffin

**Affiliations:** 1 Department of Global and Community Health, George Mason University, Fairfax, VA 22030, USA; 2 Department of Community and Population Health, Lehigh University, Bethlehem, PA 18015, USA

**Keywords:** mental health, psychological distress, substance use, university students, poor behavioral health

## Abstract

**Highlights:**

**Public health relevance—How does this work relate to a public health issue?**

- Behavioral health issues are of major concern among youth.
- Experiences of discrimination and/or microaggressions are critical social determinants of mental health.

**Public health significance—Why is this work of significance to public health?**

- University students experience a substantial burden of mental health problems.
- Students with minoritized identities face unique combinations of stressors, including those associated with experiencing discrimination and/or microaggressions on university campuses.

**Public health implications—What are the key implications or messages for practitioners, policy makers and/or researchers in public health?**

- Intersectional factors shaping the behavioral health of high-risk and minority students are nuanced.
- Prevention strategies on university campuses should focus on resilience building among those most at-risk for poor behavioral health.

**Abstract:**

Experiences of discrimination and/or microaggressions may negatively affect mental health among university students. We assessed the association between experiences of discrimination, microaggressions, and combined exposure on poor behavioral health (PBH) among university students in Spring 2023 (*N* = 45,386) using cross-sectional data from the American College Health Association’s National College Health Assessment III. PBH (an index of severe psychological distress and substance use risk) was reported by 42.9% of students. More than half of sexual and gender minority (SGM) youth reported PBH, and had a high prevalence of discrimination and microaggressions. Among racial/ethnic groups, Black/African American students had the highest prevalence of experiences of discrimination and microaggressions. Minoritized groups who experienced discrimination or microaggressions consistently reported a higher prevalence of PBH compared to their counterparts not reporting these experiences; the opposite pattern was observed among cisgender, heterosexual, and White participants. In the logistic regression models, experiences of both discrimination and microaggressions were associated with an over 2-fold increase in odds of PBH, controlling for demographic variables, compared to those experiencing neither. Interaction effects revealed that experiences of microaggressions did not consistently and differentially predict PBH across subgroups of minority youth. Efforts to increase resilience on university campuses may improve behavioral health.

## 1. Introduction

Behavioral health refers to “a state of mental, emotional, and social well-being or behaviors and actions that affect wellness” [1]. Behavioral health issues may manifest in several ways among youth, including mental health challenges, suicidal ideation, eating disorders, conduct problems, and substance use [2,3]. Among youth, university students are a critical population that face unique stressors that increase their risk for behavioral health issues. In university, students experience challenges managing academic demands, navigating the transition to independence from their families, and balancing work and family responsibilities [4], resulting in students reporting poor behavioral health compared to the general population. Thus, it is important to examine the risk factors and facilitators of behavioral health among students, as the university years are often marked by the onset or progression of mental health and substance use concerns [4]. In this study, we explore poor behavioral health (PBH) as comorbid psychological distress and substance use among university students [5].

Psychological distress, which are unpleasant mental and physical symptoms associated with mood fluctuations, is highly prevalent among students on university campuses [6,7,8,9]. For example, the National College Health Assessment American College Health Assessment (NCHA-ACHA) survey found that the prevalence of serious psychological distress among undergraduate students was 21% [10]. Notably, distress prevalence rates differ across demographic subgroups of students. Rates are typically highest among students who are female, white, and identified as non-binary (38.5%) [10]. Further, other research has shown that psychological distress and other mental health issues are elevated among females [11] and sexual and gender minority (SGM) students [12]. Studies have also shown mixed findings in mental health disparities among university students across racial and ethnic group categories, with some reporting worsening mental health among racial and ethnic minority groups (Asian American, Latinx, Multiracial students) [13] and others reporting no differences [14] when compared to their non-Hispanic White counterparts. Research in the general population has found higher rates of psychological distress among minoritized populations due to several social determinants of health factors [15,16]. Therefore, it is plausible that students with minoritized identities may be at greatest risk for mental health issues, including psychological distress due to negative experiences associated with their identities such as experiences of microaggressions, discrimination or a lack of belonging on university campuses [17,18,19].

Poor mental health may predispose students to other risky behaviors and vice versa as a means of self-medication. Particularly, severe psychological distress has been linked to worse substance use outcomes among university students. For example, studies have found that severe psychological distress may increase high-risk alcohol dependence and intermediate-to-severe drug use [20,21,22,23]. On the other hand, other evidence finds that substance use may increase the risk of developing mental health symptoms [5]. Since studies have shown that higher substance use patterns may be more prevalent among students with minoritized identities [24], research exploring comorbid mental health problems and substance among individuals with minoritized identities is needed [25,26]. This research is particularly important as students with minoritized identities may face greater risk for poor behavioral health due to a host of social and identity-related factors.

Social stressors, including financial strain [27,28], academic performance pressure [29], and loneliness [30,31], may be more prevalent among minoritized groups, increasing their risk for PBH. The minority stress theory suggests that the impact of social stressors may be particularly impactful on the mental health of individuals of minoritized identities [32]. Most significantly, these groups may be more prone to identity-related stressors, such as experiences of discrimination and microaggressions [33,34]. Discrimination refers to the “unfair or prejudicial treatment” of certain groups [35], while microaggressions are defined as “subtle abuses” based on minoritized identity [34,36,37]. According to recent evidence, the prevalence of past-year experiences of microaggressions among university students was 16.8%, while discrimination was 10.4% [37]. Experiences of discrimination and/or microaggressions are more common among students with minoritized identities, including racial, gender, and sexual orientation [38,39,40]. Research suggests that university students who identify as People of Color, Latinxs, women, SGM, and other minority groups experience the highest rates of discrimination [41,42,43] and microaggressions [36,44,45]. Experiences of discrimination usually elicit a heightened stress response effect, which may make its impact on mental health significantly stronger than that of microaggressions [41,46]. However, the differences in impact are not well understood, with a meta-analysis suggesting possible context-related differences across various minoritized identities [47]. Racial microaggressions may have worse mental health outcomes among Black women and contribute to psychological distress among Asian and Hispanic Americans [36,47]. Further, sexual orientation microaggressions may have a stronger association with negative outcomes compared to discrimination. Additionally, racial discrimination may be associated with higher rates of anxiety and psychological distress compared to microaggressions. More research is needed to understand these differences in the context of PBH. Moreover, research suggests a strong correlation between discrimination and microaggressions, suggesting the need to examine the compounded effect of these experiences on PBH.

While individual experiences of discrimination and microaggressions have been linked to worse mental health outcomes, studies suggest that these experiences also lead to increased problematic substance use [43,44], with these outcomes occurring at disproportionate rates for youth and students with minoritized identities. For example, higher racial discrimination, sexual orientation microaggressions, and SGM victimization have been linked to substance use problems among these minoritized groups on university campuses [40,42,43,45]. Existing research amplifies the need for university campuses to address acts of discrimination and microaggressions that target minoritized students in order to combat their detrimental implications on behavioral health. Further, there is a need for a multipronged approach that addresses experiences of discrimination and microaggressions in the context of co-occurring mental health and substance use. Less research has focused on how discrimination and microaggressions contribute to mental health and substance use comorbidity among those most at risk on university campuses. This study aims to fill that gap. The goals of this study were to (1) assess the prevalence of experiences of discrimination, microaggressions, and reported PBH among university students; (2) examine the unique and combined association between experiences of discrimination and microaggression and PBH; and (3) examine the groups most at risk for PBH due to experiences of discrimination and/or microaggressions. We hypothesize that the effects of discrimination and/or microaggressions are universal, but may have a particularly negative effect on PBH among minoritized groups.

## 2. Materials and Methods

### 2.1. Data Source and Sample

We used cross-sectional data from the third American College Health Association National College Health Assessment (ACHA-NCHA III) survey of post-secondary institutions across the United States. This survey includes data from undergraduate students at 125 institutions (*N* = 55,292) in Spring 2023 [48]. The ACHA-NCHA survey focuses on key topics, including mental health, substance use, and sexual health on university campuses. Inclusion criteria for this study were full-time undergraduate students between the ages of 18–24, who were enrolled in four-year U.S. institutions. The total analytic sample for this study was 45,386. We excluded part-time students and those outside the typical university age range to better align our sample with the traditional undergraduate population commonly examined in the literature. Student self-reported data were de-identified, and this analysis was deemed exempt from human subjects review by the George Mason University Institutional Review Board (IRB) per federal guidelines. Table 1 describes the demographic characteristics of the sample. The sample was predominantly female (66.6%), heterosexual (68%) with a mean age of 20 (Standard Deviation [SD] = 1.38, and over half of the population identified as White (55.3%).

### 2.2. Measures

#### 2.2.1. Demographic Variables

Gender identity was initially reported across several categories, including male, female, trans woman, trans man, genderqueer, agender, genderfluid, intersex, and non-binary. For analysis purposes, gender was recoded into three groups: male, female, and transgender and gender non-conforming (TGNC; which included all other gender identities). Sexual orientation was categorized as straight/heterosexual, bisexual, gay, lesbian, pansexual, queer, and questioning. We dichotomized sexual orientation into heterosexual and non-heterosexual (representing all other orientations). Race and ethnicity categories included Asian or Asian American, Black or African American, Hispanic or Latino/a/x, White, Mixed (Multiracial/Biracial), and Other. The “Other” category encompassed American Indian or Native Alaskan, Middle Eastern/North African (MENA) or Arab Origin, and Native Hawaiian or Other Pacific Islander. In the logistic regression, gender was recoded as TGNC = 1 and cisgender = 0; sexual orientation was recoded as non-heterosexual = 1 and heterosexual = 0; and the race and ethnicity variables were also dichotomized.

#### 2.2.2. Severe Psychological Distress

The six-item Kessler Psychological Distress Scale (K6) is a validated instrument used to assess risk for nonspecific serious mental illness over the past month [49,50]. Respondents were asked to respond to the following questions: “During the past 30 days, how often did you feel (1) nervous, (2) hopeless, (3) restless or fidgety, (4) so depressed that nothing could cheer you up, (5) that everything was an effort, and (6) worthless.” Response options were on a five-point Likert scale from “All of the time” (4) to “None of the time” (0). Individual scores were added together to yield total scores ranging from 0 to 24, with higher scores indicating worse psychological distress. A score of 0 to 4 indicates no or minimal distress, a score of 5 to 12 indicates moderate psychological distress, and a score of 13 or more indicates severe psychological distress. To compute the index variable, only scores of 13 or more were included. Those who scored 13 or higher were coded as severe psychological distress = 1, while those who scored less than 13 were coded as severe psychological distress = 0.

#### 2.2.3. WHO ASSIST

The World Health Organization (WHO) Alcohol, Smoking, and Substance Involvement Screening Test (ASSIST) is an 8-item questionnaire designed to screen for alcohol, cannabis, and other illicit substances. ASSIST generates a Substance Specific Involvement Score (SSIS) for each substance, ranging from 0–39 [51]. Higher scores reflect a higher level of risk associated with substance use. SSISs of 0–10 indicate low risk, 11–26 indicate moderate risk, and scores of 27 or greater indicate a high risk for alcohol, cannabis, and other illicit substance use dependence. To compute the index variable, only scores of 11 or higher for alcohol, cannabis, and other illicit substances were included. Those who scored 11 or higher were coded as moderate to high risk = 1, while those who scored less than 11 were coded as moderate to high risk = 0.

#### 2.2.4. Poor Behavioral Health (PBH)

For our outcome variable, PBH, we computed an index variable that represented reported severe psychological distress (measured by the K6) and one or more experiences of moderate to high use of alcohol, cannabis, and other illicit substances (measured by the WHO ASSIST). Those who scored 13 or higher on the K6 and 11 or higher for alcohol, cannabis, and other illicit substances were coded as PBH = 1, while those who scored less than 13 on the K6 and those who scored less than 11 for alcohol, cannabis, and other illicit substances were coded as PBH = 0. This outcome variable was operationalized in this way because students who experience severe psychological distress and are at high risk for substance use may be particularly vulnerable and at highest risk for poor social and academic functioning [20,21,22,23].

#### 2.2.5. Experiences of Discrimination and Microaggressions

Experiences of discrimination in the past 12 months were assessed using a one-item measure. Respondents were asked to answer yes/no to the following questions related to discrimination: “I experienced discrimination directed at me (the unjust or prejudicial treatment of a person based on the group, class, or category to which the person is perceived to belong).”

Experiences of microaggressions in the past 12 months were assessed using a one-item measure. Respondents were asked to answer yes/no to the following questions related to microaggressions: “I experienced microaggression(s) directed at me (a subtle but offensive comment or action directed at a minority or other non-dominant group, whether intentional or unintentional, that reinforces a stereotype).”

In the logistic regression model, we treated past 12-month experiences of discrimination and microaggressions separately as dichotomous variables, coded 1 = Yes and 0 = No. However, we also created a composite variable that examined the co-occurrence of discrimination and microaggressions to test the hypothesis that the cumulative effect of these exposures would be strongly associated with the study outcomes [36]. For the composite variable, we coded 1 = Yes for responses indicating both experiences of discrimination and microaggressions, and 0 = No for responses indicating no experience of discrimination or microaggressions.

### 2.3. Data Analysis

All analyses were done using the Statistical Package for the Social Sciences (SPSS) software version 28. We generated descriptive statistics to assess the prevalence of experiences of discrimination, microaggressions, and reported PBH among university students. We then examined the prevalence of PBH among groups who experienced discrimination and microaggression. We subsequently tested the association between gender, sexual orientation, race or ethnicity, experiences of discrimination and microaggression in the past 12 months, and PBH, using Logistic Regression. First, we explored the association between experiences of discrimination and microaggressions on PBH controlling for gender, sexual orientation and race or ethnicity. In this model, we tested the association for discrimination only, microaggressions only, and both simultaneously to assess their independent and combined effects on PBH. To examine the groups most at risk for PBH due to experiences of discrimination and microaggression, we included two-way interaction terms between discrimination and microaggressions and gender, sexual orientation, and race or ethnicity. Results estimating the strength of associations are reported using adjusted odds ratios with 95% confidence intervals.

## 3. Results

### 3.1. Prevalence of PBH

PBH was reported by 42.9% of students in the sample (Table 1). A higher proportion of TGNC (64.6%), non-heterosexual (58.4%), and Multi/Biracial students reported PBH. In our sample (Table 2), about 11 percent reported experiencing discrimination, while about 19 percent reported experiencing microaggressions. Individuals who identified as TGNC (27% & 45.8%), Black or African American (24.5% & 38.6%), and non-heterosexual (17.7% & 32%) reported experiencing higher rates of discrimination and microaggressions, respectively, compared to other groups.

Among students who identified as TGNC, those who experienced discrimination and microaggressions reported higher rates of PBH than those who did not experience discrimination or microaggressions (Table 3). Across gender categories, females reported the highest rates of PBH due to experiences of discrimination and microaggressions compared to males and TGNC students. Similarly, students who identified as non-heterosexual and experienced discrimination (57% vs. 39.9%) and microaggressions (60.6% vs. 36.4%) had a higher prevalence of reported PBH than those who did not experience discrimination and microaggressions. Across racial or ethnic categories, students who experienced discrimination and microaggressions had a higher prevalence of reported PBH compared to their counterparts who did not experience discrimination and microaggressions. Asian or Asian American, Black or African American, Hispanic, Other Race, and Multi/Biracial students who experienced discrimination or microaggressions had a higher prevalence of reported PBH compared to their counterparts who did not experience discrimination and microaggressions. Students who identified as White had the highest prevalence of reported PBH compared to other racial or ethnic groups, whether or not they experienced discrimination or microaggressions.

In summary, across gender, race/ethnicity, and sexual orientation categories, minoritized groups who experienced discrimination or microaggressions consistently reported higher prevalence of PBH compared to their counterparts who did not experience discrimination or microaggressions; the opposite pattern was observed among majority groups (cisgender, Whites, heterosexual). These patterns support our hypothesis that discrimination and microaggressions play a more important role in PBH among minoritized groups compared to majority groups.

### 3.2. Discrimination and Microaggressions: Unique and Combined Effects

In the logistic regression model (Table 4), after controlling for gender, sexual orientation, and race or ethnicity, students who experienced discrimination (OR = 1.73) or microaggressions (OR = 1.67) had higher odds of reporting PBH compared to those who did not experience them. However, we found that those who reported experiencing discrimination *and* microaggression had over twice the odds of reporting PBH after controlling for demographic characteristics (OR = 2.37).

### 3.3. Interaction Between Experiences of Discrimination and Microaggressions and Gender

**Figure 1:**
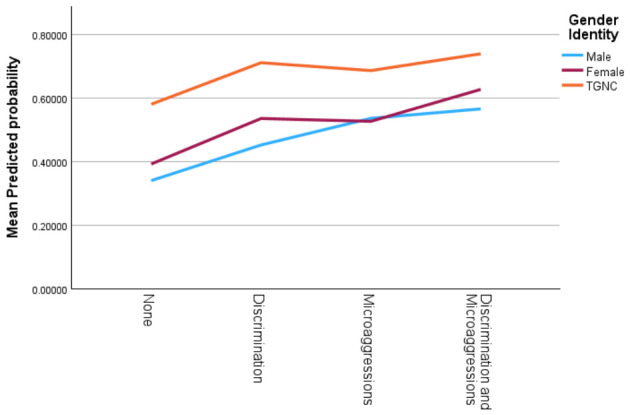
Mean predicted probability of poor behavioral health by gender identity and exposure to discrimination and microaggressions. Significant associations are reported in Table 5.

In the logistic regression model (Table 5), female (OR = 1.25) and TGNC students (OR = 2.68) had significantly greater odds of reporting PBH compared to males, with TGNC students showing the largest disparity (Figure 1). Experiences of discrimination, microaggressions, and combined exposure were each significantly associated with PBH, with microaggressions posing a higher risk than discrimination and the combined exposure having the strongest risk association. Significant negative interactions emerged for female × microaggressions (OR = 0.77) and TGNC × microaggressions (OR = 0.71), indicating that the marginal effect of microaggression exposure was attenuated for these groups.

### 3.4. Interaction Between Experiences of Discrimination and Microaggressions and Sexual Orientation

**Figure 2:**
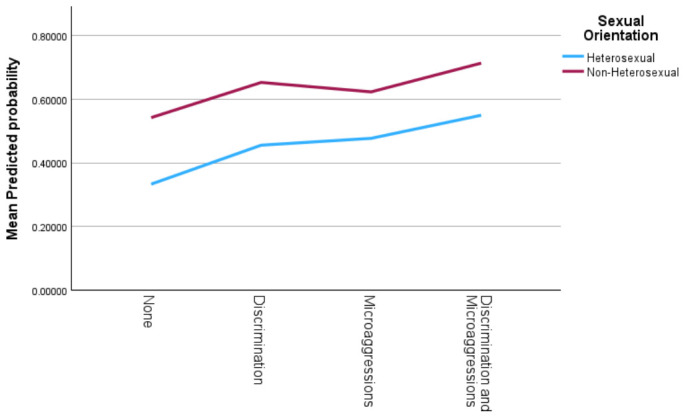
Mean predicted probability of poor behavioral health by sexual orientation and exposure to discrimination and microaggressions. Significant associations are reported in Table 6.

Non-heterosexual students had more than double the odds of reporting PBH compared to heterosexual peers (OR = 2.37; Table 6). Discrimination, microaggressions, and combined exposure were each significantly associated with PBH, with microaggressions posing a higher risk than discrimination and the combined exposure having the strongest risk association (Figure 2). A significant attenuating interaction was observed between non-heterosexual identity and microaggressions (OR = 0.76).

### 3.5. Interaction Between Experiences of Discrimination and Microaggressions and Race or Ethnicity

Significant differences emerged across racial and ethnic groups (Tables S1–S7; Figures S1–S6). White (OR = 1.25) and Multi/Biracial students (OR = 1.21) had higher odds of reporting PBH than their comparison groups, whereas Black (OR = 0.67) and Asian/Asian American students (OR = 0.64) had significantly lower odds. Hispanic ethnicity and “Other” race showed no significant main effects. Similar to previous findings, discrimination, microaggressions, and combined exposure were robustly associated with PBH, with microaggressions associated with greater risk than discrimination and combined exposure producing the largest effects (ORs: 2.75–3.07) across all racial/ethnic categories. The interactions for White × microaggressions (OR = 1.19) and Asian × discrimination and microaggressions (OR = 1.21) were amplifying, while Multi/Biracial × microaggressions (OR = 0.83) were attenuating. No significant interactions were observed for Black, Hispanic, or “Other” race students.

## 4. Discussion

This study explored the prevalence and association between experiences of discrimination, microaggressions, and reported PBH among university students, examining the groups most at risk for PBH. Findings showed that PBH was reported by almost half of the sample, meaning that co-morbid psychological distress and substance use dependence are significant issues among university students. We found that the prevalence of reported PBH was highest among students who identified as TGNC, non-heterosexual, and multi/biracial compared to other groups. This is consistent with extant literature, showing similar trends in the same population [52,53,54]. These groups of students may be exposed to multiple risk factors for PBH outside of experiences of discrimination and microaggressions. In the logistic regression model, findings showed that students who identified as TGNC, compared to females and males, and non-heterosexuals, compared to heterosexuals, had the greatest odds of PBH. Though TGNC students were the fewest in the sample, these disparities in PBH are notable. More work is needed to support SGM individuals on college campuses, including the need for more inclusive policies and interventions that improve mental health and reduce substance use dependence as a coping mechanism [55]. Females who experienced discrimination or microaggressions had a higher prevalence of PBH compared to their counterparts, consistent with other research [56]. In the logistic regression, we found that females had higher odds of PBH compared to males. Research shows that females may be more likely to have internalizing disorders compared to males [57], suggesting the need for targeted interventions that consider the role of gendered stressors on PBH.

One in ten students experienced discrimination, and almost one in five students experienced microaggressions. We examined the unique and combined association between experiences of discrimination and microaggression and PBH, and found that both exposures independently predicted stronger odds of PBH. After controlling for demographic characteristics, students who experienced both discrimination and microaggressions had the highest odds of reported PBH compared to those who experienced either. This suggests that the combined effect of experiencing both discrimination and microaggressions leads to worse outcomes for students. However, those who experienced discrimination had higher odds of PBH compared to those who experienced microaggressions, consistent with extant research [46]. Interestingly, in the individual models for each of the demographic groups, we found that microaggressions consistently outweighed the impact of discrimination in these models. This suggests that while overt discrimination may be generally more consequential, the impact of microaggressions, which are more frequent and chronic in nature, cannot be overlooked, given that their cumulative exposure may produce sustained internalizing symptoms [47], which constitute core components of PBH.

Our study sought to identify the groups most at risk for PBH due to experiences of discrimination and/or microaggressions. We found that the prevalence of PBH was higher among minoritized groups who experienced discrimination or microaggressions compared to those who did not, as initially hypothesized. In the logistic regression, we observed a weaker association between experiences of microaggressions and PBH among TGNC, female, and non-heterosexual students. While we found that Asian or Asian Americans were less likely than non-Asians to report PBH, the relationship between experiences of both discrimination and microaggressions and PBH was stronger among Asian or Asian Americans compared to non-Asians. This finding is supported by extant literature showing that experiences of discrimination or microaggressions among Asian American students were associated with poor psychological adjustment [36]. Findings also showed that students who identified as Multi/Biracial had higher odds of reported PBH compared to non-Multi/Biracial students, respectively. We did not observe an interaction effect for discrimination or among other racial or ethnic minoritized groups in this study. It is plausible that students of minoritized status often face chronic, systemic discrimination from a young age and may develop specific resilience strategies or community support systems to buffer the impact on their mental health.

White participants had among the highest rates of PBH across race/ethnic groups, and reported the lowest rates of discrimination and microaggressions. The interaction effects with Whites showed that microaggressions had an outsized impact on PBH among the small proportion of Whites that reported them. One explanation for this finding may be that the experience of discrimination and microaggressions among White participants was a novel experience, and that these individuals do not have the cultural resilience related to coping with chronic discrimination that other minority groups may have. It is also plausible that students who identified as White in this sample may have multiple intersecting identities, including sexual orientation and gender, that put them in the numerical minority. Thus, future studies examining experiences of discrimination and/or microaggressions should consider non-demographic-related identity factors that place students in the numeric minority and the impact of these factors on PBH.

The strengths of our study include the use of a large national dataset and the use of measures with established psychometric properties. However, our study has some limitations. First, the use of cross-sectional data limited our ability to make causal inferences between the predictor and outcome variables. The NCHA-ACHA data is not a representative sample of university students and is self-reported data, which is subject to selection bias, recall bias, and underreporting of sensitive behaviors. One plausible explanation for the absence of intersectional effects is the lack of a comprehensive measure of discrimination and microaggressions that fully captures the nuanced experiences of these groups [58,59,60]. Moreover, identity-related factors are more nuanced, as minoritized students often face multiple stressors simultaneously, which may attenuate the observed effects of discrimination and microaggressions. Therefore, studies need to include comprehensive survey instruments when collecting data on these important topics to accurately measure each construct. Further research, particularly qualitative studies, would provide deeper insight into the contextual and intersectional factors shaping the behavioral health of high-risk and minority students. Information on types of discrimination was not collected, and though we stratify across gender, sexual orientation, and race and ethnicity, we cannot speak to the forms of discrimination that may affect poor behavioral outcomes. Lastly, the data may not fully capture intersectional experiences, which are highly relevant for understanding discrimination and microaggressions.

## 5. Conclusions

Our study highlights the significant burden of PBH among university students, with 42.9 percent of the sample meeting the criteria for severe distress and substance use risk. We also demonstrate how discrimination and microaggressions can contribute to PBH across demographic subgroups of students. These findings indicate that tailored, comprehensive campus interventions are needed to address discrimination and foster resilience, including culturally responsive mental health services, campus-wide anti-discrimination policies and training, peer support and mentorship programs, and skills-based interventions that strengthen coping and stress management.

## Figures and Tables

**Table 1:** Demographic Characteristics and Distribution of Reported Poor Behavioral Health among University Students in Spring 2023 by Demographic Characteristics (*N* = 45,386).

			Reported Poor Behavioral Health (*N* = 19,472, 42.9%)	
	*N*	%	*N*	%
Gender				
Male	11,763	25.9	4347	37
Female	30,229	66.6	12,976	42.9
TGNC	3017	6.6	1950	64.6
Race or Ethnicity				
Asian	6846	15	2443	35.7
Black or African American	1992	4.4	799	40.1
Hispanic	4643	10.2	1988	42.8
Other Race ^†^	819	1.8	365	44.6
Multi/Biracial	4758	10.5	2285	48
White	25,112	55.3	11,015	43.9
Sexual Orientation				
Heterosexual	30,880	68	11,072	35.9
Non-Heterosexual	14,057	31	8213	58.4

^†^ Includes American Indian or Native Alaskan, Middle Eastern/North African (MENA) or Arab Origin, Native Hawaiian or Other Pacific Islander Native; TGNC: Transgender and Gender Non-conforming.

**Table 2:** Distribution of Experiences of Discrimination and Microaggressions among University Students in Spring 2023 by Demographic Characteristics (*N* = 45,386).

	Experienced Discrimination (*N* = 5115, 11.3%)		Experienced Microaggressions (*N* = 8424, 18.6%)	
	*N*	%	*N*	%
Gender				
Male	1030	8.8	1384	11.8
Female	3182	10.6	5530	18.4
TGNC	810	27	1376	45.8
Race or Ethnicity				
Asian or Asian American	999	14.7	1630	24
Black or African American	485	24.5	763	38.6
Hispanic	671	14.5	995	21.5
Other Race ^†^	173	21.2	221	27.1
Multi/Biracial	705	14.9	1309	27.6
White	1860	7.4	3179	12.7
Sexual Orientation				
Heterosexual	2560	8.3	3822	12.4
Non-Heterosexual	2479	17.7	4482	32

^†^ Includes American Indian or Native Alaskan, Middle Eastern/North African (MENA) or Arab Origin, Native Hawaiian or Other Pacific Islander Native.

**Table 3:** Prevalence of Reported Poor Behavioral Health among University Students in Spring 2023 by Experiences of Discrimination and Microaggression and Demographic Characteristics (*N* = 45,386).

	Reported Poor Behavioral Health							
	Experienced Discrimination		No Experiences of Discrimination		Experienced Microaggressions		No Experiences of Microaggressions	
	*N*	%	*N*	%	*N*	%	*N*	%
Gender								
Male	532	17.6	3797	23.5	759	15.6	3570	24.9
Female	1899	62.8	11,045	68.2	3127	64.3	9814	68.4
TGNC	594	19.6	1348	8.3	980	20.1	964	6.7
Race or Ethnicity								
Asian or Asian American	550	18.7	1877	11.8	843	17.7	1586	11.3
Black or African American	277	9.4	519	3.3	415	8.7	379	2.7
Hispanic	407	13.9	1579	9.9	598	12.6	1386	9.8
Other Race ^†^	103	3.5	259	1.6	125	2.6	237	1.7
Multi/Biracial	455	15.5	1824	11.5	786	16.5	1495	10.6
White	1146	39	9840	61.9	1989	41.8	8998	63.9
Sexual Orientation								
Heterosexual	1305	43	9735	60.1	1929	39.4	9110	63.6
Non-Heterosexual	1731	57	6455	39.9	2963	60.6	5225	36.4

^†^ Includes American Indian or Native Alaskan, Middle Eastern/North African (MENA) or Arab Origin, Native Hawaiian or Other Pacific Islander Native. Note: Values represent prevalence rates for each subgroup.

**Table 4:** Adjusted Associations Between Experiences of Discrimination and Microaggressions and Reported Poor Behavioral Health among University Students in Spring 2023 (*N* = 45,386).

	B	S.E.	Wald χ^2^	OR	95% C.I.
Discrimination	0.55	0.05	108.01 ***	1.73	1.56, 1.92
Microaggressions	0.51	0.03	240.71 ***	1.67	1.57, 1.78
Discrimination and Microaggressions	0.86	0.04	442.03 ***	2.37	2.18, 2.56
Female ^†^	0.11	0.02	22.06 ***	1.12	1.07, 1.17
TGNC ^†^	0.37	0.05	58.71 ***	1.45	1.32, 1.59
Non-Heterosexual *	0.73	0.02	966.66 ***	2.07	1.98, 2.17
Black ^‡^	−0.34	0.05	46.17 ***	0.71	0.64, 0.78
Asian ^‡^	−0.40	0.03	176.82 ***	0.67	0.63, 0.71
Hispanic ^‡^	−0.09	0.03	6.75 **	0.92	0.86, 0.98
Other race ^‡^	−0.08	0.08	1.28	0.92	0.79, 1.06
Multi/Biracial ^‡^	0.02	0.03	0.35	1.02	0.96, 1.09

^†^ Reference group is Cisgender; * Reference group is Heterosexual; ^‡^ Reference group is White; *** *p* < 0.001; ** *p* < 0.01.

**Table 5:** Associations Between Gender, Experiences of Discrimination and Microaggressions and Reported Poor Behavioral Health among University Students in Spring 2023 (*N* = 45,386).

	B	S.E.	Wald χ^2^	OR	95% C.I.
Female ^†^	0.22	0.03	80.36 ***	1.25	1.19, 1.32
TGNC ^†^	0.99	0.06	301.19 ***	2.68	2.40, 3.00
Discrimination	0.47	0.10	23.73 ***	1.60	1.32, 1.93
Microaggressions	0.81	0.07	119.67 ***	2.24	1.94, 2.59
Discrimination and Microaggressions	0.93	0.09	113.74 ***	2.53	2.13, 2.99
FemalexDiscrimination	0.11	0.12	0.90	1.12	0.89, 1.40
FemalexMicroaggressions	−0.26	0.08	10.18 **	0.77	0.65, 0.90
FemalexDiscrimination and Microaggressions	0.03	0.10	0.09	1.03	0.85, 1.25
TGNCxDiscrimination	0.11	0.21	0.25	1.11	0.73, 1.68
TGNCxMicroaggressions	−0.35	0.12	8.22 **	0.71	0.56, 0.90
TGNCxDiscrimination and Microaggressions	−0.21	0.13	2.41	0.81	0.62, 1.06

^†^ Reference group is Males; *** *p* < 0.001; ** *p* < 0.01.

**Table 6:** Associations Between Sexual Orientation, Experiences of Discrimination and Microaggressions and Reported Poor Behavioral Health among University Students in Spring 2023 (*N* = 45,386).

	B	S.E.	Wald χ^2^	OR	95% C.I.
Non-Heterosexual ^†^	0.86	0.02	1191.14 ***	2.37	2.26, 2.49
Discrimination	0.52	0.06	68.48 ***	1.67	1.48, 1.89
Microaggressions	0.60	0.04	192.51 ***	1.83	1.68, 1.99
Discrimination and Microaggressions	0.89	0.05	271.89 ***	2.44	2.19, 2.71
Non-HeterosexualxDiscrimination	−0.05	0.11	0.25	0.95	0.77, 1.17
Non-HeterosexualxMicroaggressions	−0.27	0.06	18.14 ***	0.76	0.68, 0.87
Non-HeterosexualxDiscrimination and Microaggressions	−0.15	0.08	3.79	0.86	0.74, 1.00

^†^ Reference group is Heterosexual; *** *p* < 0.001.

## Data Availability

Restrictions apply to the availability of these data. Data were obtained from ACHA-NCHA (access date: 28 February 2024) and are available at https://www.acha.org/ncha/data-results/data-access-published-literature/ with the permission of ACHA-NCHA. Persons interested in using the data are encouraged to submit proposals to the administrator for access to the data.

## Supplementary Materials

The following supporting information can be downloaded at: https://www.mdpi.com/article/10.3390/ijerph23060776/s1, Table S1. Distribution of Reported Poor Behavioral Health among University Students in Spring 2023 by Demographic Characteristics (*N* = 45,386). Table S2. Associations Between White Race, Experiences of Discrimination and Microaggressions and Reported Poor Behavioral Health among University Students in Spring 2023 (*N* = 45,386); Figure S1. Mean predicted probability of poor behavioral health by White racial identity and exposure to discrimination and microaggressions. Significant associations are reported in Table S2; Table S3. Associations Between Multi/Biracial Identity, Experiences of Discrimination and Microaggressions and Reported Poor Behavioral Health among University Students in Spring 2023 (*N* = 45,386); Figure S2. Mean predicted probability of poor behavioral health by Multi/Biracial identity and exposure to discrimination and microaggressions. Significant associations are reported in Table S3; Table S4. Associations Between Black Race, Experiences of Discrimination and Microaggressions and Reported Poor Behavioral Health among University Students in Spring 2023 (*N* = 45,386); Figure S3. Mean predicted probability of poor behavioral health by Black racial identity and exposure to discrimination and microaggressions. Significant associations are reported in Table S4; Table S5. Associations Between Asian/Asian American Race, Experiences of Discrimination and Microaggressions and Reported Poor Behavioral Health among University Students in Spring 2023 (*N* = 45,386); Figure S4. Mean predicted probability of poor behavioral health by Asian/Asian American racial identity and exposure to discrimination and microaggressions. Significant associations are reported in Table S5; Table S6. Associations Between Hispanic Ethnicity, Experiences of Discrimination and Microaggressions and Reported Poor Behavioral Health among University Students in Spring 2023 (*N* = 45,386); Figure S5. Mean predicted probability of poor behavioral health by Hispanic ethnicity and exposure to discrimination and microaggressions. Significant associations are reported in Table S6; Table S7. Associations Between “Other” Race, Experiences of Discrimination and Microaggressions and Reported Poor Behavioral Health among University Students in Spring 2023 (*N* = 45,386); Figure S6. Mean predicted probability of poor behavioral health by “Other” racial identity and exposure to discrimination and microaggressions. Significant associations are reported in Table S7.

## Abbreviations

The following abbreviations are used in this manuscript:

CI	Confidence Interval
IRB	Institutional Review Board
K6	Kessler Psychological Distress Scale
MENA	Middle Eastern/North American
NCHA-ACHA	National College Health Assessment American College Health Assessment
NCHA-ACHA III	National College Health Assessment American College Health Assessment III
OR	Odds Ratio
PBH	Poor Behavioral Health
SGM	Sexual gender minority
SPSS	Statistical Package for Social Sciences
SSIS	Substance Specific Involvement Score
WHO	World Health Organization
