# Supplementary material for: The Prevalence of Poor Behavioral Health Among University Students by Gender, Sexual Orientation, and Racial Identity: The Role of Discrimination and Microaggressions
Source: Int J Environ Res Public Health. 2026 Jun 9;23(6):776. doi: 10.3390/ijerph23060776 (PMC13299882; doi:10.3390/ijerph23060776)
Supplement: Supplementary file 1 [file ijerph-23-00776-s001.zip › ijerph-4204516-supplementary.pdf]

The Prevalence of Poor Behavioral Health Among University Students by Gender, Sexual Orientation, and Racial Identity: The Role of Discrimination and Microaggressions

Tolulope M. Okuneye[1],Andrew J. Godley[1], Elaine C. Russell[1], Lisa L. Lindley[2], Kenneth W. Griffin[1]

[1]Department of Global and Community Health, George Mason University, Fairfax, VA 22030, USA

[2]Department of Community and Population Health, Lehigh University, Bethlehem, PA 18015, USA

Supplementary Materials

Table S1. Distribution of Reported Poor Behavioral Health among University Students in Spring 2023 by Demographic Characteristics (*N*=45,386)

	Reported Poor Behavioral Health (*N*=19472, 42.9%)		No Reported Poor Behavioral Health (*N*=25914, 57.1%)	
	N	%	N	%
Gender				
Male	4347	37	7416	63
Female	12976	42.9	17253	57.1
TGNC	1950	64.6	1067	35.4
Race or Ethnicity				
Asian or Asian American	2443	35.7	4403	64.3
Black or African American	799	40.1	1193	59.9
Hispanic	1988	42.8	2655	57.2
Other Race†	365	44.6	454	55.4
Multi/Biracial	2285	48	2473	52
White	11015	43.9	14097	56.1
Sexual Orientation				
Heterosexual	11072	35.9	19808	64.1
Non-Heterosexual	8213	58.4	5844	41.6
Experienced Discrimination	3083	60.3	2032	40.7
Experienced Microaggressions	4956	58.8	3468	41.2

†Includes American Indian or Native Alaskan, Middle Eastern/North African (MENA) or Arab Origin, Native Hawaiian or Other Pacific Islander Native

Table S2. Associations Between White Race, Experiences of Discrimination and Microaggressions and Reported Poor Behavioral Health among University Students in Spring 2023 (*N*=45,386)

	B	S.E.	Wald χ^2	OR	95% C.I.
White†	0.22	0.02	94.50***	1.25	1.20, 1.31
Discrimination	0.59	0.07	67.52***	1.80	1.57, 2.07
Microaggressions	0.65	0.04	232.47***	1.91	1.76, 2.08
Discrimination and Microaggressions	1.12	0.05	551.95***	3.07	2.79, 3.37
WhitexDiscrimination	0.03	0.10	0.08	1.03	0.84, 1.26
WhitexMicroaggressions	0.17	0.06	7.63**	1.19	1.05, 1.35
WhitexDiscrimination and Microaggressions	-0.09	0.08	1.13	0.92	0.78, 1.08

*†Reference group is Non-White; ***p < 0.001; **p <0.01*

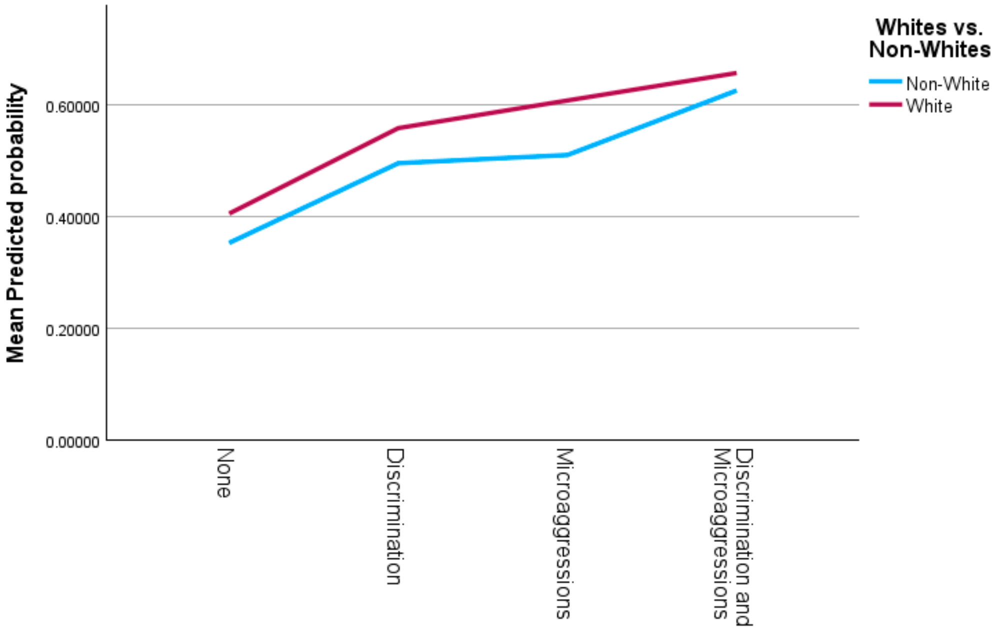

Figure S1. Mean predicted probability of poor behavioral health by White racial identity and exposure to discrimination and microaggressions. Significant associations are reported in Table S2.

Table S3. Associations Between Multi/Biracial Identity, Experiences of Discrimination and Microaggressions and Reported Poor Behavioral Health among University Students in Spring 2023 (*N*=45,386)

	B	S.E.	Wald χ^2	OR	95% C.I.
Multi/Biracial†	0.19	0.04	26.22***	1.21	1.12, 1.30
Discrimination	0.57	0.05	110.02***	1.76	1.59, 1.96
Microaggressions	0.70	0.03	428.05***	2.01	1.88, 2.15
Discrimination and Microaggressions	1.02	0.04	609.64***	2.77	2.55, 3.00
Multi/BiracialxDiscrimination	0.03	0.17	0.04	1.03	0.75, 1.43
Multi/BiracialxMicroaggressions	-0.19	0.09	4.58*	0.83	0.70, 0.98
Multi/BiracialxDiscrimination and Microaggressions	-0.02	0.11	0.05	0.98	0.79, 1.20

*†Reference group is Non-Multi/Biracial; ***p < 0.001; *p <0.05*

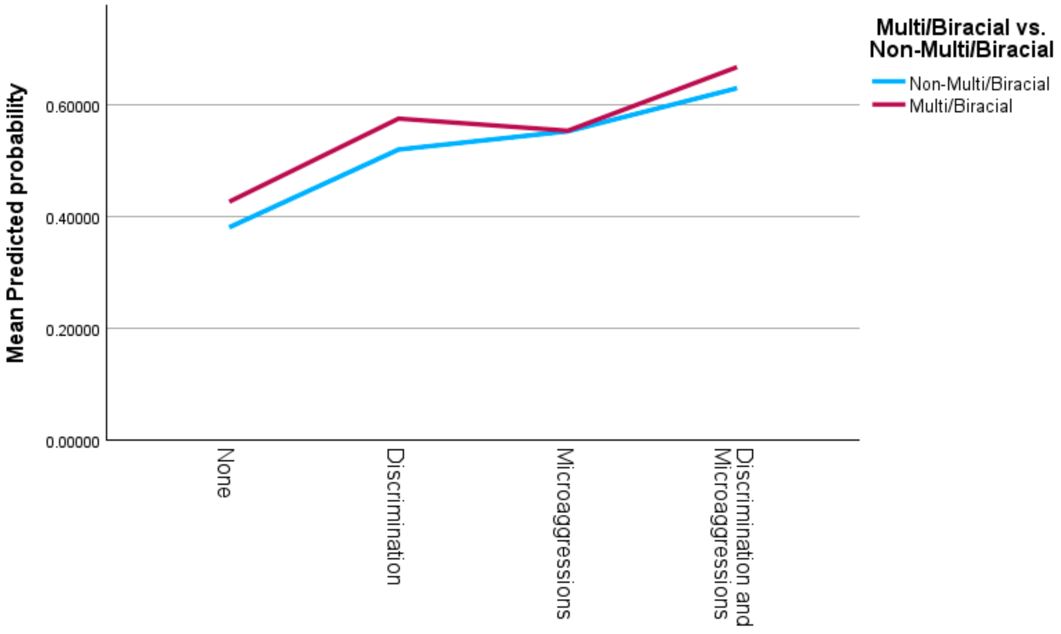

Figure S2. Mean predicted probability of poor behavioral health by Multi/Biracial identity and exposure to discrimination and microaggressions. Significant associations are reported in Table S3.

Table S4. Associations Between Black Race, Experiences of Discrimination and Microaggressions and Reported Poor Behavioral Health among University Students in Spring 2023 (*N*=45,386)

	B	S.E.	Wald χ^2	OR	95% C.I.
Black†	-0.40	0.07	36.33***	0.67	0.59, 0.76
Discrimination	0.57	0.05	119.48***	1.78	1.60, 1.97
Microaggressions	0.69	0.03	458.33***	1.99	1.87, 2.12
Discrimination and Microaggressions	1.04	0.04	656.95***	2.82	2.61, 3.05
BlackxDiscrimination	0.15	0.22	0.47	1.16	0.75, 1.79
BlackxMicroaggressions	0.11	0.13	0.80	1.12	0.87, 1.43
BlackxDiscrimination and Microaggressions	0.21	0.13	2.73	1.24	0.96, 1.59

*†Reference group is Non-Black; ***p < 0.001*

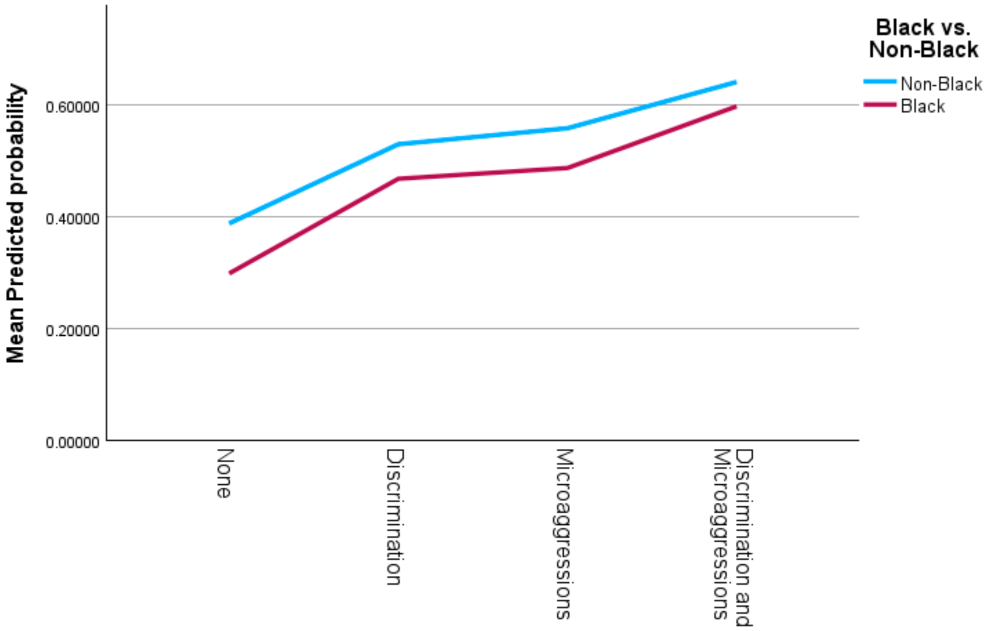

Figure S3. Mean predicted probability of poor behavioral health by Black racial identity and exposure to discrimination and microaggressions. Significant associations are reported in Table S4.

Table S5. Associations Between Asian/Asian American Race, Experiences of Discrimination and Microaggressions and Reported Poor Behavioral Health among University Students in Spring 2023 (***N***=45,386)

	B	S.E.	Wald χ^2	OR	95% C.I.
Asian/Asian American†	−0.45	0.03	179.97***	0.64	0.60, 0.68
Discrimination	0.58	0.06	105.87***	1.78	1.60, 1.99
Microaggressions	0.71	0.03	422.27***	2.03	1.90, 2.17
Discrimination and Microaggressions	1.02	0.04	562.08***	2.78	2.56, 3.03
Asian/Asian AmericanxDiscrimination	0.07	0.14	0.26	1.07	0.82, 1.40
Asian/Asian AmericanxMicroaggressions	−0.02	0.08	0.04	0.98	0.84, 1.15
Asian/Asian AmericanxDiscrimination and Microaggressions	0.19	0.09	4.40*	1.21	1.01, 1.46

*†Reference group is Non-Asian; ***p < 0.001; *p <0.05*

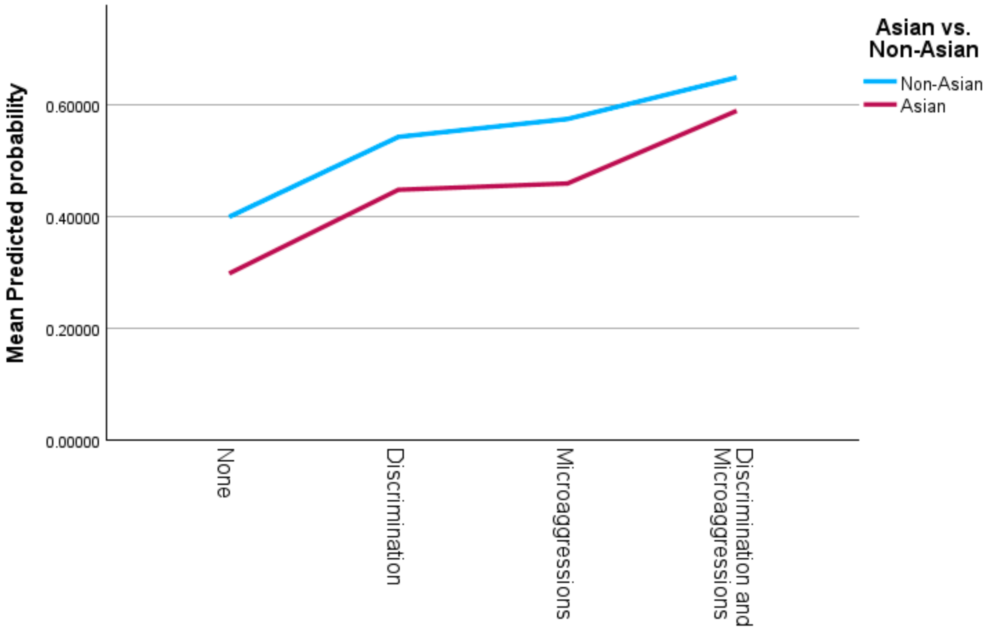

Figure S4. Mean predicted probability of poor behavioral health by Asian/Asian American racial identity and exposure to discrimination and microaggressions. Significant associations are reported in Table S5.

Table S6. Associations Between Hispanic Ethnicity, Experiences of Discrimination and Microaggressions and Reported Poor Behavioral Health among University Students in Spring 2023 (***N***=45,386)

	B	S.E.	Wald χ^2	OR	95% C.I.
Hispanic†	−0.06	0.04	2.44	0.94	0.88, 1.01
Discrimination	0.58	0.05	111.19***	1.78	1.60, 1.98
Microaggressions	0.67	0.03	417.45***	1.96	1.84, 2.09
Discrimination and Microaggressions	1.01	0.04	612.10***	2.75	2.54, 2.30
HispanicxDiscrimination	−0.02	0.15	0.02	0.98	0.73, 1.31
HispanicxMicroaggressions	0.08	0.10	0.63	1.08	0.89, 1.31
HispanicxDiscrimination and Microaggressions	0.13	0.11	1.43	1.14	0.92, 1.43

†*Reference group is Non-Hispanic; ***p < 0.001*

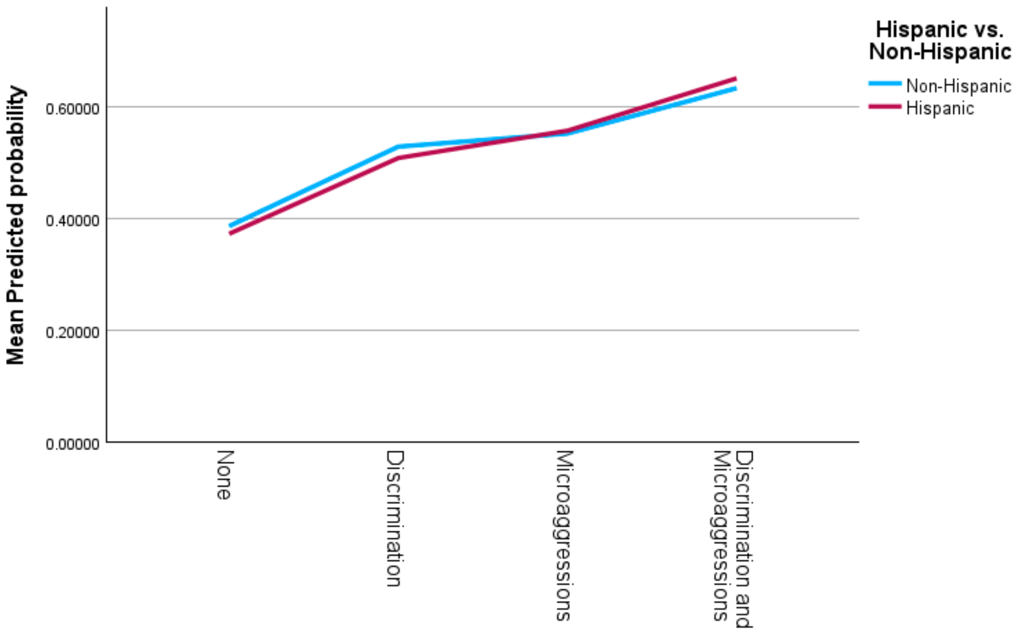

Figure S5. Mean predicted probability of poor behavioral health by Hispanic ethnicity and exposure to discrimination and microaggressions. Significant associations are reported in Table S6.

Table S7. Associations Between "Other" Race, Experiences of Discrimination and Microaggressions and Reported Poor Behavioral Health among University Students in Spring 2023 (***N***=45,386)

	B	S.E.	Wald χ^2	OR	95% C.I.
Other†	0.02	0.09	0.03	1.02	0.85, 1.21
Discrimination	0.58	0.05	124.28***	1.79	1.61, 1.98
Microaggressions	0.69	0.03	480.76***	1.99	1.87, 2.12
Discrimination and Microaggressions	1.02	0.04	702.14***	2.78	2.58, 3.00
OtherxDiscrimination	-0.20	0.28	0.50	0.82	0.48, 1.42
OtherxMicroaggressions	-0.35	0.21	2.66	0.70	0.46, 1.07
OtherxDiscrimination and Microaggressions	0.07	0.22	0.09	1.07	0.69, 1.64

*"Other" category includes American Indian or Native Alaskan, Middle Eastern/North African (MENA) or Arab Origin, Native Hawaiian or Other Pacific Islander Native; †Reference group is other race outside American Indian or Native Alaskan, Middle Eastern/North African (MENA) or Arab Origin, Native Hawaiian or Other Pacific Islander Native; ***p < 0.001*

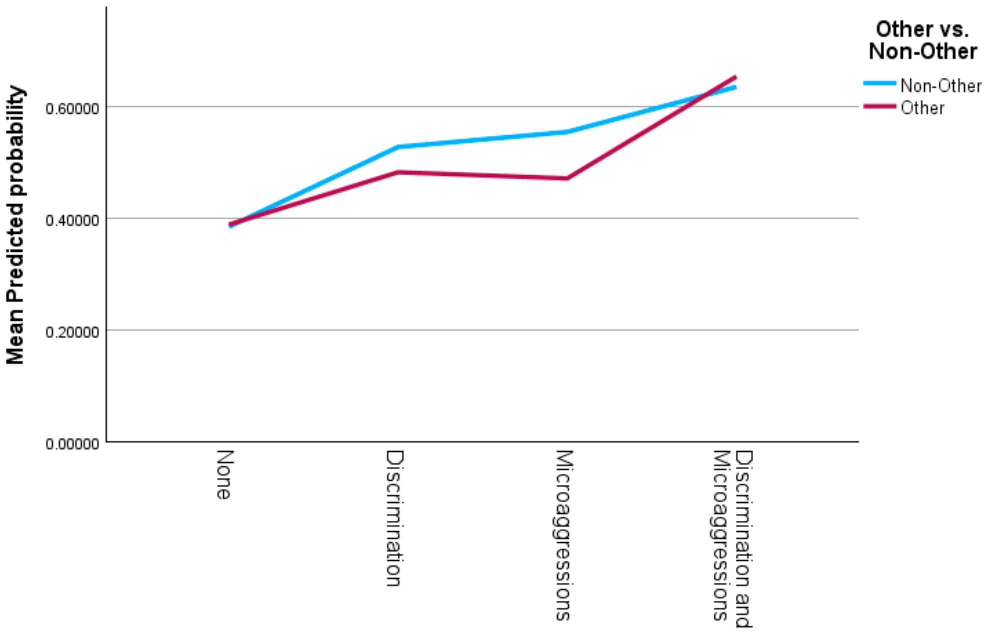

Figure S6. Mean predicted probability of poor behavioral health by "Other" racial identity and exposure to discrimination and microaggressions. Significant associations are reported in Table S7.
